# Establishing a database for sickle cell disease patient mapping and survival tracking: The sickle pan-african research consortium Nigeria example

**DOI:** 10.3389/fgene.2022.1041462

**Published:** 2022-11-04

**Authors:** Obiageli Nnodu, Anazoeze Madu, Reuben Chianumba, Hezekiah Alkali Isa, Isaac Olanrewaju, Samuel Osagie, Nash Oyekanmi, Raphael Zozimus Sangeda, Annemie Stewart, Victoria Nembaware, Jack Morrice, Mario Jonas, Gaston Mazandu, Ambroise Wonkam, Olumide Owolabi

**Affiliations:** ^1^ Centre of Excellence for Sickle Cell Disease Research and Training (CESRTA), University of Abuja, Abuja, Nigeria; ^2^ Department of Haematology and Blood Transfusion, University of Abuja, Abuja, Nigeria; ^3^ Department of Haematology and Immunology, College of Medicine, University of Nigeria, Enugu, Nigeria; ^4^ Department of Computer Science, University of Abuja, Abuja, Nigeria; ^5^ ITMS Unit, University of Abuja, Abuja, Nigeria; ^6^ H3Africa Bioinformatics Network (H3ABioNet) Node, Centre for Genomics Research and Innovation, Abuja, Nigeria; ^7^ Department of Pharmaceutical Microbiology, Muhimbili University of Health and Allied Sciences, Dar Es Salaam, Tanzania; ^8^ UCT Clinical Research Centre, Faculty of Health Sciences, University of Cape Town, Cape Town, South Africa; ^9^ Computational Biology Division, IDM, Faculty of Health Sciences, University of Cape Town, Cape Town, South Africa; ^10^ Division of Human Genetics, Department of Pathology, Faculty of Health Sciences, University of Cape Town, Cape Town, South Africa; ^11^ Faculty of Health Sciences, Department of Pathology, Division of Human Genetics, University of Cape Town, Cape Town, South Africa; ^12^ Department of Medicine, Faculty of Health Sciences, University of Cape Town, Cape Town, South Africa

**Keywords:** database, REDCap, sickle cell disease, SPARCO, Nigeria, registry

## Abstract

**Background:** The Sickle Pan-African Research Consortium (SPARCO) and Sickle Africa Data Coordinating Center (SADaCC) were set up with funding from the US National Institute of Health (NIH) for physicians, scientists, patients, support groups, and statisticians to collaborate to reduce the high disease burden and alleviate the impact of Sickle Cell Disease (SCD) in Africa. For 5 years, SPARCO and SADaCC have been collecting basic clinical and demographic data from Nigeria, Tanzania, and Ghana. The resulting database will support analyses to estimate significant clinical events and provide directions for targeting interventions and assessing their impacts.

**Method:** The Nigerian study sited at Centre of Excellence for Sickle Cell Disease Research and Training (CESRTA), University of Abuja, adopted REDCap for online database management. The case report form (CRF) was adapted from 1,400 data elements adopted by SPARCO sites. It captures 215 data elements of interest across sub-sites, i.e., demographic, social, diagnostic, clinical, laboratory, imaging, and others. These were harmonized using the SADaCC data dictionary. REDCap was installed on University of Abuja cloud server at https://www.redcap.uniabuja.edu.ng. Data collected at the sites are sent to CESRTA for collation, cleaning and uploading to the database.

**Results:** 7,767 people living with sickle cell disease were enrolled at 25 health institutions across the six zones in Nigeria with 5,295 having had at least one follow-up visit with their clinical data updated. They range from 44 to 1,180 from 3 centers from South East, 4 from South, 5 from South West, 8 from North Central, 4 in North West and 3 in the North East. North West has registered 1,383 patients, representing 17.8%; North East, 359 (4.6%); North Central, 2,947 (37.9%); South West, 1,609 (20.7%); South, 442 (5.7%) and South East, 1,027 patients (13.2%).

**Conclusion:** The database is being used to support studies including analysis of clinical phenotypes of SCD in Nigeria, and evaluation of Hydroxyurea use in SCD. Reports undergoing review in journals have relied on the ease of data access in REDCap. The database is regularly updated by batch and individual record uploads while we are utilizing REDCap’s in-built functions to generate simple statistic.

## Introduction

Sickle cell disease (SCD) is an inherited blood disorder arising from mutations in the gene coding for the β globin chain of haemoglobin. This leads to pathogenetic processes culminating in vessel occlusion and progressive ischemia and tissue necrosis. The disease is inherited in a simple Mendelian pattern (Piel et al., 2013) and affects more than 5 million people in Sub-Saharan Africa (SSA). About 150,000 children are born with this condition annually in this region. The incidence of SCD in Nigeria varies from 2% to 3% as reported by various investigators in the different regions of the Country (Grosse et al., 2011).

The disease is marked by acute exacerbations of pain and anemia warranting frequent hospital or clinic admissions and blood transfusion. Chronic complications such as osteonecrosis, leg ulcers, pulmonary hypertension and renal failure may occur. This requires the presence of appropriately trained medical personnel to provide care at various levels of healthcare. This has huge implications on the health economics and fiscal deployment in both adults and children (Baker et al., 2015). There are approximately 111.91 cases of homozygous S (SS) per 100,000 newborns globally, while Africa has a rate of 1,125.49 (Wastnedge et al., 2018). The disease burden of people living with SCD is currently estimated to be approximately 250 million people worldwide, with the majority living in the rural areas of Sub-Saharan Africa (SSA) and with limited access to health care.

The socio-economic impact of the disease on affected persons and families is massive with frequent morbidity associated with a high mortality rate (Olatunya et al., 2015; Nnodu et al., 2021). Health budgeting and intervention planning requires a good estimate of disease burden and patient mapping in order to effectively link patients to care and track other occurrences of significant disease-related events.

An extensive database is also necessary for effective and comprehensive documentation of the natural course of the disease and its complications in the different unique socio-cultural backgrounds found in Africa. The establishment and maintenance of a simple database would provide real time as well as retrospective information on the clinical state of patients across the country. This will also offer a platform for the estimation of significant clinical events including frequency of vaso occlusive crisis, transfusion needs, frequency of complications, pattern of drug prescription as well as survival. These will provide direction targeting interventions and assessing impact of such interventions.

The Sickle Pan-African Research Consortium (SPARCO) and Sickle Africa Data Coordinating Center (SADaCC) consists of physicians, scientists, patients support groups and statisticians who work together to reduce the disease burden and alleviate the impact of disease. These primary objectives are to be achieved by establishing a research database, carrying out research aimed at alleviating disease burden and reducing its impact on already affected persons. SPARCO and SADaCC have over the past 5 years accumulated and synchronized basic clinical and demographic data from Nigeria, Tanzania and Ghana. Challenges abound in a resource-limited setting but the benefits are enormous with regards to limiting waste and–optimizing care.

## Databases and other computer based systems

Databases were designed for persistent and integrated storage of data, allowing concurrent access to it by many users. They are collections of records related by referential integrity. Thus, a database is an organized collection of structured data meant to serve many applications with minimum redundancy (Elmasri and Navathe, 2017). Database technology helps to alleviate many of the problems associated with conventional file organization methods, including data duplication, inflexibility and difficulties associated with online access by users.

Databases, in greatly aiding the availability and searchability of data, have become and indispensable tool for research in medical and other fields. For example, well-designed biological databases have become a powerful tool in biological research (Dong et al., 2015). Also, it is now well established that keeping electronic medical records leads to dramatic improvements in the level of care provided by health institutions. This was shown in a 2006 study on electronic medical records (Akor et al., 2018). The study showed, among others, that EMR facilitates easy access to medication administration records, sharing of consultation reports, and decreased transit time of test results by reducing the time taken to deliver paper versions.

Other developments in computing and computer technologies that have benefitted medical research include Data/text mining, Computer modeling. Medical imaging/anomaly detection and Machine learning. Applications of these techniques have been discussed in the literature (Hand et al., 2001; Lei and Karniadakis, 2013; Solanki, 2014; Khalaf et al., 2016). The use of Pattern Recognition techniques in medicine have also been studied (Benamrane et al., 2005; Bacardit et al., 2014). An interesting case of the application of text and data mining in SCD research is the development of an information exploration system, Dragon Exploration System for SCD (DESSCD), which aims to promote the easy exploration of SCD related data. The system processed 419,612 MEDLINE abstracts retrieved from a PubMed query using SCD-related keywords. The processed SCD-related data was then made available *via* the DESSCD web query interface that enables information retrieval using specified concepts, keywords and phrases, and the generation of inferred association networks and hypotheses (Essack, 2013).

## REDCap and electronic data capture

One tool currently used by researchers in many countries to manage research data collections is the Research Electronic Data Capture (REDCap) application. REDCap is a mature, secure web application designed to support data capture and managing online databases. While REDCap can be used to collect virtually any type of data, it is specifically geared to support data capture for research studies. The application allows users to build and manage online surveys and databases quickly and securely, and is currently in production use or development build-status for more than 290,000 projects with over 398,000 users spanning numerous research focus areas across the consortium (Harris et al., 2019).

There are many other Electronic Data Capture software that are being used in medical research; some of these are Catalyst Web Tools, OpenClinica, IBM Clinical Development and Videoc; REDCap has been found to compare well with some of these other systems (Franklin et al., 2011). It has even been suggested that the strength of REDCap as it pertains to its use in medical research is that it is primarily a clinical research database built by clinical researchers specifically for clinical research (Patridge and Bardyn, 2018). REDCap is a secondary survey tool that provides an easy-to-use interface and several desirable features for data collection, management, analysis, and reporting (Harris et al., 2009; Harris et al., 2019).

Projects are self-sufficient and workflow-based; the focus is on collecting data and exporting it to statistical programs and other data analysis software. REDCap is designed to provide a secure environment so that research teams can collect and store highly sensitive information. It is a flexible tool that can run on multiple operating systems such as Linux, UNIX, Windows, and Mac. We deployed REDCap at CESRTA, with an administrator and 4 data clerks who manage the recording of data into the database. The administrator for the system is a certified cloud infrastructure engineer from the University of Abuja’s IT unit. The Principal Investigator (PI) and Data Manager were also assigned administrator roles. The users are verified members of the research team; the Site Lead determines the level of access and privileges granted to each user by the administrators.

## Methodology

The study site was based in the Centre of Excellence for Sickle Cell Disease Research and Training (CESRTA), University of Abuja. The subjects are patients recruited into various research projects at CESRTA (https://cesrta.uniabuja.edu.ng/). Data was collected from consenting patients attending sickle cell clinics using case report forms (CRFs) designed for the project after ethical approval. The soft copy of the CRFs were produced on Excel sheets and shared with all the collaborating sites in Nigeria. This was preceded by a meeting of all the site leads who are doctors who run the adult and paediatric sickle cell clinics all over Nigeria and were available to be part of SPARCO. In these meetings data managers/collectors were trained on how to fill the CRFs and also to input the data electronically. The data collected with the Excel sheet are regularly sent for central collation at CESRTA where it is then batch uploaded into the REDCap database.

There are two studies currently ongoing on the REDCAP platform, 1) the SPARCO description of phenotype and 2) the survival study from the newborn screening. For the SPARCO phenotype study, we had a local care report form which was used to collect full demographic and clinical data from patients attending the SCD clinics at SPARCO centers across the country. This was then used to develop the CRF for enrollment of patients into the SPARCO registry. The hard copy CRF forms were deployed to the SPARCO Nigeria Sites for patient enrollment. Site leads were brought to CESRTA for a data management workshop and training on the use of REDCap. Feedback was received and used to modify the first CRF, standardizing it and making it possible to separate features of past medical history, presenting symptoms and monitor follow-up data. The CRF was designed to achieve full clinical phenotyping and to obtain laboratory information as they become available. It included demographic, social, diagnostic, clinical, laboratory, imaging, and research data elements, all totaling 125 variables (“field name”), to be included in the database. These variables were harmonized using the SADaCC data dictionary; trainings were also conducted by the Big Data Analytics team in order to assist the team members harmonize the data elements in line with the SCD Ontology. Before implementation in REDCap. REDCap was installed on the University of Abuja dedicated cloud server by the University’s server Administrator/Webmaster at https://www.redcap.uniabuja.edu.ng alongside requisite applications such as MySQL database server and PHP. An Administrator, who can add users to the software and assign them permissions, was then designated. Users can create new projects or be assigned roles by project owners.

Creating a new project is done at the +New Project tab after logging in to REDCap. When a user creates a new project, the project is automatically set in development status, allowing the user to edit the project and test it before uploading real data. Once a project has moved to production status, it can no longer be edited.

The REDCap administrator created a project for our site with the record auto-numbering feature enabled. After thoroughly testing the project setup and being found satisfactory, it was moved to Production Status, which made it available online for duly authorized users.

Data tables were created in the Project to adequately represent all the data being gathered. Such Tables included: Basic demographics of the screened population, results of screening, contact numbers, exact location, names of schools and nearest primary health care centres. For those with sickle haemoglobin, SS or SC, only basic patient data (demographics), routine clinic visits, acute care visits, hospital admissions and laboratory studies are collected. Each table was developed with relevant data elements suggested by the requirements analysis and was translated into user interface forms and field-tested at the data collection sites. Changes were made in the design of the Tables based on the results of the field test. All these were created on the REDCap project with appropriate form interfaces for data capture. At CESRTA, the data collected from the screening points were initially prepared in MS Excel worksheets and uploaded in comma-separated (csv) format following the Data Dictionary template. The use of mobile devices were later introduced, allowing for secure on-site clinical use as well as the collection and transmission of research data to the online project. In addition, individual data records are sometimes entered directly as the need arises. All the submitted data are subjected to quality checks by the data management team.

## Results

Data was obtained from 27 centers made up of 25 tertiary health institutions, 2 secondary and one private hospital currently engaged in SPARCO Nigeria patient enrollment. Table 1 shows the centres across the country that are currently enrolling patients on the SPARCO platform.

**TABLE 1:** Enrolment by SPARCO centers across Nigeria.

Hospital name abbreviation	Institution’s full name	Geopolitical zone	Number
UMTH	University of Maidugiri Teaching Hospital	North East	200
FTH, GOMBE	Federal Teaching Hospital Gombe		78
FMC Yola	Federal Medical Centre, Yola		81
BDTH	BARAU DIKKO TEACHING HOSPITAL KADUNA	North West	243
ABUTH	Ahmadu Bello University Teaching Hospital, Zaria		599
AKTH	Aminu Kano Teaching Hospital		291
FMC, BK	Federal Medical Centre, Birnin Kebbi		250
JUTH	Jos University Teaching Hospital	North Central	679
UATH	University of Abuja Teaching Hospital, Gwagwalada		1,180
FMC KEFFI	Federal Medical Centre Keffi		378
NHA, P	National Hospital Abuja		316
NGH	General Hospital Nyanya		202
NHA, A	National Hospital Abuja, Adult		99
ZMC	ZANKLI MEDICAL CENTRE, Abuja		49
MGH	Maitama General Hospital		44
UNTH	University of Nigeria Teaching Hospital, Enugu	South East	426
AEFUTHA	Federal Teaching Hospital Abakaliki		280
NAUTH	Nnamdi Azikiwe University Teaching Hospital		321
UCH	University College Hospital, Ibadan	South West	678
OAUTH, WGH	Obafemi Awolowo University Teaching Hospital, WGH		551
LASUTH	Lagos State University Teaching Hospital		101
OAUTH, C	Obafemi Awolowo University Teaching Hospital College		144
LUTH	Lagos University Teaching Hospital		135
FMC ASABA	Federal Medical Asaba	South	111
RSUTH	River State University Teaching Hospital		163
ISTH	ISTH Irrua		99
UUTH	University of Uyo Teaching Hospital		69
	Total		7,767

**FIGURE 1:**
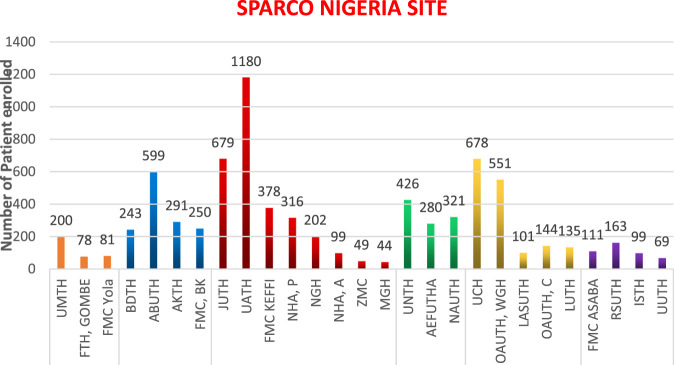
Enrolment by SPARCO Nigeria sites.

A total of 7,767 people living with SCD have been enrolled into the SPARCO Nigeria data platform and another 5,295 have had at least one follow up visit with their clinical data updated. This represents an estimated 0.001% of the entire population of sickle cell patients in Nigeria. The number of recruited patients range from 44 to 1,180 and included 3 centers from South East, 4 from South, 5 from South West, 8 from North Central, 4 in North West and 3 in the North East. As at the time of this report, the North West had registered 1,383 patients, representing 17.8%, North East, 359 patients (4.6%), North Central, 2,947 patients (37.9%), South West, 1,609 patients (20.7%), South, 442 patients (5.7%) and South East, 1,027 patients (13.2%). This is shown in Figure 1.

From the basic analysis done on the data with the REDCap analysis tools, the following were observed:

Age: the age of patients’ ranges from 0 to 64 years, with a mean value of 16.58 and median value of 16 years

**FIGURE 2:**
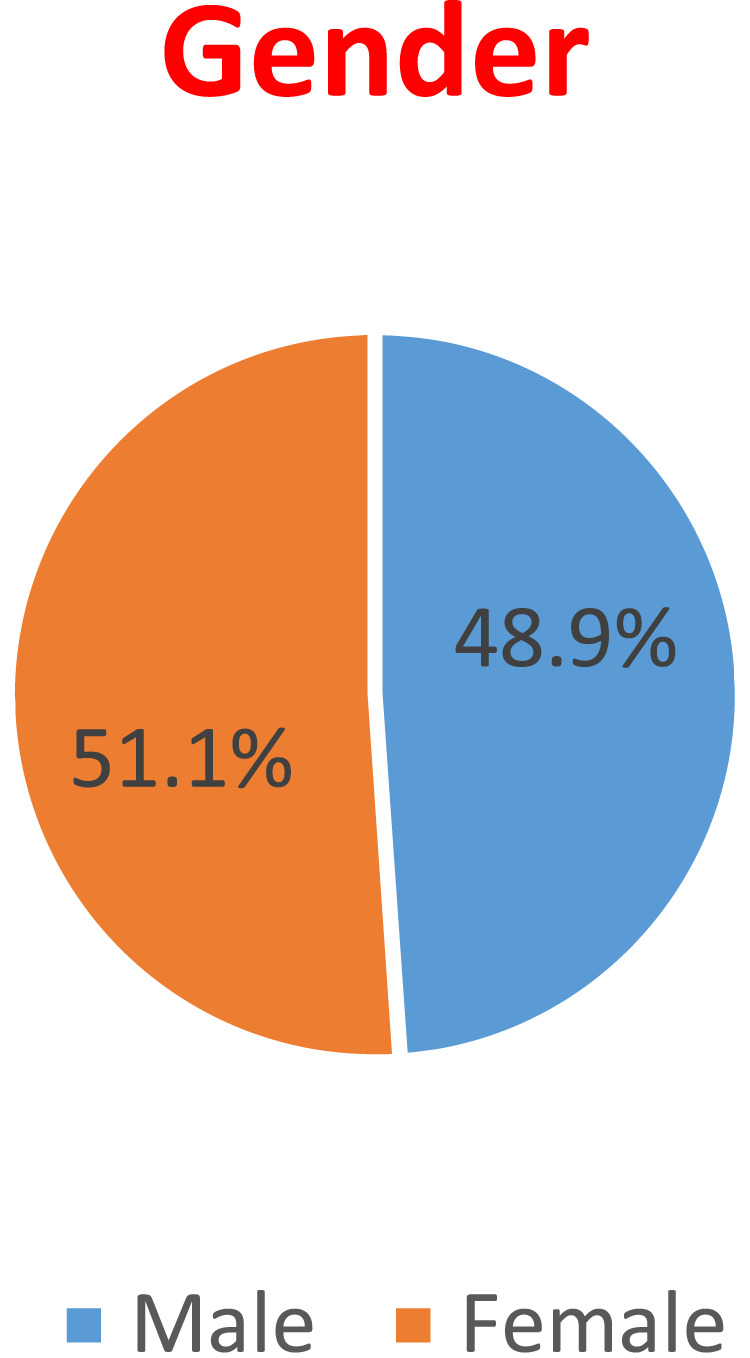
Patient distribution by Sex.

Gender: 48.9% of the patients are Males, while 51.1% are Females. This displayed in Figure 2.

**FIGURE 3:**
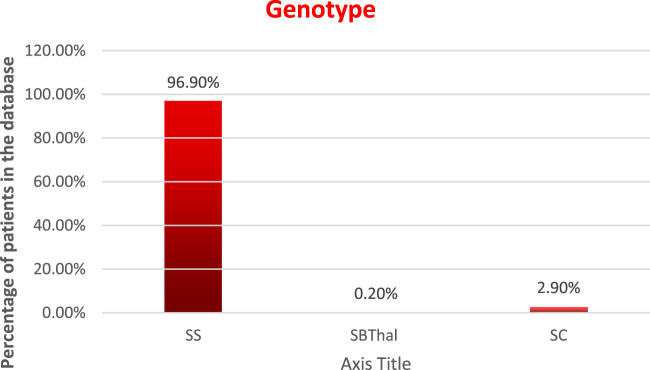
Distribution of patients by Genotype.

Genotype: the distribution of the patients by genotype is as follows, and is displayed in Figure 3.

HbSS—96.9%
HbSBThal—0.2%
HbSC—2.9%, and
HbAS—0%

It could also be observed that, though the data was collected at 27 centres from across the country, all the States of the Nigerian Federation were represented in the data set. The database could thus be said to be generally representative of the Nigerian population.

## Discussion

Using REDCap features as described, we had designed data tables to store all the data elements defined in the Nigeria-specific Case Reporting Form (CRF) and SPARCO data dictionary. The online availability of the REDCap database makes it convenient for the Team members to access the database for data research, insertion, updates, viewing and reporting according to the access privileges of each member. This has been demonstrated by a preliminary analysis of the clinical phenotypes of SCD in Nigeria which was published (Isa et al., 2020) and a study on the evaluation of Hydroxyurea use in SCD (Chianumba et al., 2022). Other completed studies with submitted manuscripts undergoing review in different journals have relied heavily on the ease of access to data in the REDCap project. Data upload is also regularly done both by batch upload and individual record insertion.

Several other studies on SCD have used REDCap for data storage and management and they report on the strengths of the software, which include rapid development of forms by technical staff with limited programming skills (Patridge and Bardyn, 2018), flexibility in data entry and analysis, efficient and secure methods for data collection, ease of use and quick turnaround, and very straightforward data exports, with exported data automatically coming in SPSS, Excel/CSV, SAS, R, and STATA formats (Franklin et al., 2011). Availability of copious amounts of training materials has also been cited as an advantage (Franklin et al., 2011). Using REDCap has aided the project’s data management function in a highly resourceful manner. From converting the CRF to representative data tables, and enforcing data quality and integrity, to executing data queries, REDCap provides the tools to effectively execute all desirable data management functions. We have also been able to utilize some of its in-built functions to generate some simple statistics on the data. To continually support security of the research data, and to maintain smooth data management operations, the University of Abuja REDCap instance has gone through a couple of upgrades resulting in the current version being used. This registry is an additional resource to the existing sickle cell databases in Africa and other parts of the world such as the Globin Research Network for Data and Discovery (GRNDaD), Sickle Cell Clinical Research and Intervention Program (SCCRIP), the Sickle Cell Data Collection (SCDC) program all in the United States. (Boye-Doe et al., 2020; Hankins. et al., 2018; CDC (Centers for Disease Control and Prevention), 2020). In the United Kingdom, some of the sickle cell registries include, the NHS Sickle Cell and Thalassaemia Screening Programme, National Haemoglobinopathy Register, Screening Wales and Cardiff Sickle Cell and Thalassaemia Centre, and Paediatric and Adult Haematology Lead Republic of Ireland. (Dormandy et al., 2018). Most of these registries collect longitudinal data and are multicentred. The SCCRIP database is a single institution registry and in addition, has retrospective data. The main objective of these registries is the evaluation of outcomes of interventions and research with a view to improving the quality of life and survival of patients with SCD.

## Conclusion

The initial challenge is that of getting like-minded researchers to agree with regards to the importance and value of available patient data on a particular disease. This required several correspondence and meetings and some funding. Another option is to rely on already existing networks, like SPARCO in this case.

Similar to the Tanzanian study reported in (Tluway and Makani, 2017), this work addresses the paucity of elaborate databases for many non-communicable diseases in Africa. The lack of cohesion and sustainability had hampered the availability of an online and broad-based data pool of sickle cell patients in Africa. Setting up such an online massive database is froth with several challenges that were dealt with in this manuscript. This manuscript will therefore serve as a guide for future data collection of a large pool of patients by researchers across several countries and on a variety of diseases.

## Data Availability

The original contributions presented in the study are included in the article/supplementary material, further inquiries can be directed to the corresponding author.
