# Supplementary material for: Establishing a database for sickle cell disease patient mapping and survival tracking: The sickle pan-african research consortium Nigeria example
Source: Front Genet. 2022 Nov 4;13:1041462. doi: 10.3389/fgene.2022.1041462 (PMC9672677; doi:10.3389/fgene.2022.1041462)
Supplement: Supplementary file 1 [file DataSheet1.PDF]

Data Dictionary Codebook

12/10/2022 10:55pm

	#	Variable / Field Name	Field Label<br>*Field Note*	Field Attributes (Field Type, Validation, Choices, Calculations, etc.)
Instrument: **Screening** (screening)				
	1	[record_id]	Research Subject ID Research ID	text
	2	[date_today]	Visit Date (dd/mm/yyyy)	text (date_dmy)
	3	[participant_number]	Participant Number<br>*Site-specific ID eg. NG20184321 for Nigeria*	text
	4	[scd_number]	SCD Number	text
	5	[hospital_name]	Hospital Name	text
	6	[medical_record_number]	Medical Record Number (MRN)	text
	7	[first_name]	First Name	text, Identifier
	8	[middle_name]	Middle Name	text, Identifier
	9	[last_name]	Last Name	text, Identifier
	10	[date_of_birth]	Date of Birth (dd/mm/yyyy)	text (date_dmy)
	11	[age]	Age	calc<br>Calculation: round(datediff([date_of_birth], [date_today],'y','dmy'),0)<br>Custom alignment: RH
	12	[gender]	Gender	radio<br>1 Male<br>2 Female<br>3 Refused<br>Custom alignment: RH
	13	[marital_status]	Marital Status	radio<br>1 Single<br>2 Married<br>3 Other<br>4 Cohabiting<br>5 Divorced<br>6 Widowed
	14	[religion]	Religion	dropdown<br>1 Christianity<br>2 Islam<br>3 African traditional beliefs<br>4 Others
	15	[tribe]	Tribe	text

	16	[state_of_origin]	State of Origin	dropdown<br>1 Abia<br>2 Adamawa<br>3 Anambra<br>4 Akwa Ibom<br>5 Bauchi<br>6 Bayelsa<br>7 Benue<br>8 Borno<br>9 Cross River<br>10 Delta<br>11 Ebonyi<br>12 Enugu<br>13 Edo<br>14 Ekiti<br>15 Gombe<br>16 Imo<br>17 Jigawa<br>18 Kaduna<br>19 Kano<br>20 Katsina<br>21 Kebbi<br>22 Kogi<br>23 Kwara<br>24 Lagos<br>25 Nasarawa<br>26 Niger<br>27 Ogun<br>28 Ondo<br>29 Osun<br>30 Oyo<br>31 Plateau<br>32 Rivers<br>33 Sokoto<br>34 Taraba<br>35 Yobe<br>36 Zamfara<br>37 Federal Capital Territory (FCT)
	17	[lga]	LGA	text
	18	[place_of_birth]	Place of Birth	text
	19	[year_of_diagnosis]	Section Header: *Diagnosis Details*<br>Year of Diagnosis	text (integer, Min: 1900, Max: 2100)
	20	[scd_test_result_ss_sbthal]	SCD Test Result (SS/SBThal/SC/CC)	radio<br>1 SS<br>2 SBThal<br>3 SC<br>4 CC<br><br>Custom alignment: RH

	21	[abo_blood_group]	ABO Blood Group	dropdown<br>1 A+<br>2 A-<br>3 B+<br>4 B-<br>5 O+<br>6 O-<br>7 AB+<br>8 AB-
	22	[using_hydroxyurea]	Section Header: *Management Details*<br>Using Hydroxyurea	radio<br>1 Yes<br>2 No<br>3 Truly unknown<br>4 Not asked / Not collected<br><br>Custom alignment: RH
	23	[using_hydroxyurea_ever]	Using Hydroxyurea (Ever)	radio<br>1 Yes<br>2 No<br>3 Truly unknown<br>4 Not asked / Not collected<br><br>Custom alignment: RH
	24	[date_of_initiation_of_hydr]	Date of Initiation of Hydroxyurea Therapy	text (date_dmy)
	25	[penicillin_v_prophylaxis]	Penicillin V (prophylaxis)	radio<br>1 Yes<br>2 No<br>3 Truly unknown<br>4 Not asked / Not collected<br><br>Custom alignment: RH
	26	[penicillin_v_prophylaxis_ever]	Penicillin V (prophylaxis) (Ever)	radio<br>1 Yes<br>2 No<br>3 Truly unknown<br>4 Not asked / Not collected<br><br>Custom alignment: RH
	27	[folic_acid]	Folic Acid	radio<br>1 Yes<br>2 No<br>3 Truly unknown<br>4 Not asked / Not collected<br><br>Custom alignment: RH

	28	[folic_acid_ever]	Folic Acid (Ever)	radio<br>1 Yes<br>2 No<br>3 Truly unknown<br>4 Not asked / Not collected<br><br>Custom alignment: RH
	29	[antimalaria_prophylaxis]	Anti Malaria Prophylaxis	radio<br>1 Yes<br>2 No<br>3 Truly unknown<br>4 Not asked / Not collected<br><br>Custom alignment: RH
	30	[antimalaria_prophylaxis_ever]	Anti Malaria Prophylaxis (Ever)	radio<br>1 Yes<br>2 No<br>3 Truly unknown<br>4 Not asked / Not collected<br><br>Custom alignment: RH
	31	[pneumococcal_vacc_uptodate]	Pneumococcal Vaccination up to date	radio<br>1 Yes<br>2 No<br>3 Truly unknown<br>4 Not asked / Not collected<br><br>Custom alignment: RH
	32	[residence]	Section Header: *Contact Details*<br>Residence	text, Identifier
	33	[street]	Street	text, Identifier
	34	[lga_residence]	LGA of Residence	text
	35	[state_of_residence]	State of Residence	text
	36	[geo_zone]	Geopolitical Zone	text
	37	[telephone1]	Telephone1	text, Identifier
	38	[telephone2]	Telephone2	text, Identifier
	39	[next_of_kin_name]	Next-of-kin Name	text, Identifier
	40	[next_of_kin_relationship]	Relationship to the Patient	text
	41	[next_of_kin_telephone_numb]	Next of Kin Telephone Number	text, Identifier
	42	[consent_obtained]	Section Header: *Consent*<br>Has the patient consented?	radio<br>1 Yes<br>2 No<br>7 Refused<br>9 Don't Know
	43	[consent_type]	Type of informed consent	checkbox<br>1 consent_type___1 Study enrollment<br>2 consent_type___2 Specimen Storage Consent<br>3 consent_type___3 Genetic Material Storage Consent<br>4 consent_type___4 Broad consent

	44	[consent_date]	Date subject signed consent	text (date_dmy)
	45	[consent_file]	Upload the consent form	file
	46	[added_by]	Added by	text<br>Field Annotation: @USERNAME @READONLY-FORM @HIDDEN
	47	[add_date]	Add Date	text (date_dmy)<br>Field Annotation: @TODAY @READONLY-FORM @HIDDEN
	48	[last_edited_by]	Last edited by	text<br>Field Annotation: @USERNAME @HIDDEN
	49	[last_edit_date]	Last edit Date	text (date_dmy)<br>Field Annotation: @TODAY @READONLY-FORM @HIDDEN
	50	[blood_transfusion]	Blood Transfusion	radio<br>1 Yes<br>2 No<br>3 Truly unknown<br>4 Not asked / Not collected
	51	[number_episodes]	Number of Episodes	text
	52	[number_of_units_transfused]<br>Show the field ONLY if:<br>[blood_transfusion] = '1'	Number of Units Transfused	text
	53	[freq_transfusion]	Frequency of Transfusion in the last year	text
	54	[date_of_last_transfusion]<br>Show the field ONLY if:<br>[blood_transfusion] = '1'	Date of Last Transfusion (dd/mm/yyyy)	text (date_dmy)
	55	[frequency_of_pain_crisis_per_year]	Frequency of Pain Crisis per Year	text (number)
	56	[other_pain_site]	Other Pain Site	text
	57	[swelling]	Swelling	radio<br>1 Yes<br>2 No<br>4 Not asked / Not collected
	58	[learning_difficulty]	Learning Difficulty	radio<br>1 Yes<br>2 No<br>4 Not asked / Not collected
	59	[hospitalized_30_days]	Hospitalized 30 days prior to visit	radio<br>1 Yes<br>2 No<br>4 Not asked / Not collected
	60	[abnormal_gait]	Abnormal GAIT	radio<br>1 Yes<br>2 No<br>4 Not asked / Not collected
	61	[difficulty_in_walking]	Difficulty in walking	radio<br>1 Yes<br>2 No<br>4 Not asked / Not collected

	62	[anaemia]	Anaemia	radio<br>1 Yes<br>2 No<br>3 Truly unknown<br>4 Not asked / Not collected
	63	[fever]	Fever	radio<br>1 Yes<br>2 No<br>3 Truly unknown<br>4 Not asked / Not collected
	64	[malaria]	Malaria	radio<br>1 Yes<br>2 No<br>3 Truly unknown<br>4 Not asked / Not collected
	65	[headache]	Headache	radio<br>1 Yes<br>2 No<br>3 Truly unknown<br>4 Not asked / Not collected
	66	[dizziness]	Dizziness	radio<br>1 Yes<br>2 No<br>3 Truly unknown<br>4 Not asked / Not collected
	67	[dizziness_ever]	Dizziness (Ever)	radio<br>1 Yes<br>2 No<br>3 Truly unknown<br>4 Not asked / Not collected
	68	[seizures]	Seizures	radio<br>1 Yes<br>2 No<br>3 Truly unknown<br>4 Not asked / Not collected
	69	[seizures_ever]	Seizures (Ever)	radio<br>1 Yes<br>2 No<br>3 Truly unknown<br>4 Not asked / Not collected
	70	[dactylitis]	Dactylitis	radio<br>1 Yes<br>2 No<br>3 Truly unknown<br>4 Not asked / Not collected

	71	[dactylitis_ever]	Dactylitis (Ever)	radio<br>1 Yes<br>2 No<br>3 Truly unknown<br>4 Not asked / Not collected
	72	[bone_pain]	Bone Pain	radio<br>1 Yes<br>2 No<br>3 Truly unknown<br>4 Not asked / Not collected
	73	[bone_pain_ever]	Bone Pain (Ever)	radio<br>1 Yes<br>2 No<br>3 Truly unknown<br>4 Not asked / Not collected
	74	[hip_pain]	Hip Pain	radio<br>1 Yes<br>2 No<br>3 Truly unknown<br>4 Not asked / Not collected
	75	[hip_pain_ever]	Hip Pain (Ever)	radio<br>1 Yes<br>2 No<br>3 Truly unknown<br>4 Not asked / Not collected
	76	[chest_pain]	Chest Pain	radio<br>1 Yes<br>2 No<br>3 Truly unknown<br>4 Not asked / Not collected
	77	[chest_pain_ever]	Chest Pain (Ever)	radio<br>1 Yes<br>2 No<br>3 Truly unknown<br>4 Not asked / Not collected
	78	[acute_chest_syndrome]	Acute Chest Syndrome	radio<br>1 Yes<br>2 No<br>3 Truly unknown<br>4 Not asked / Not collected
	79	[acute_chest_syndrome_ever]	Acute Chest Syndrome (Ever)	radio<br>1 Yes<br>2 No<br>3 Truly unknown<br>4 Not asked / Not collected

	80	[leg_ulcer]	Leg Ulcer	radio<br>1 Yes<br>2 No<br>3 Truly unknown<br>4 Not asked / Not collected
	81	[leg_ulcer_ever]	Leg Ulcer (Ever)	radio<br>1 Yes<br>2 No<br>3 Truly unknown<br>4 Not asked / Not collected
	82	[osteonecrosis]	Osteonecrosis (Avascular Necrosis)	radio<br>1 Yes<br>2 No<br>3 Truly unknown<br>4 Not asked / Not collected
	83	[osteonecrosis_ever]	Osteonecrosis (Avascular Necrosis) (Ever)	radio<br>1 Yes<br>2 No<br>3 Truly unknown<br>4 Not asked / Not collected
	84	[difficulty_in_breathing]	Difficulty in breathing	radio<br>1 Yes<br>2 No<br>3 Truly unknown<br>4 Not asked / Not collected
	85	[difficulty_in_breathing_ever]	Difficulty in breathing (Ever)	radio<br>1 Yes<br>2 No<br>3 Truly unknown<br>4 Not asked / Not collected
	86	[exertional_dyspnaea]	Exertional Dyspnaea	radio<br>1 Yes<br>2 No<br>3 Truly unknown<br>4 Not asked / Not collected
	87	[exertional_dyspnaea_every]	Exertional Dyspnaea (Ever)	radio<br>1 Yes<br>2 No<br>3 Truly unknown<br>4 Not asked / Not collected
	88	[pulmonary_hypertension]	Pulmonary Hypertension	radio<br>1 Yes<br>2 No<br>3 Truly unknown<br>4 Not asked / Not collected

	89	[pulmonary_hypertension_ever]	Pulmonary Hypertension (Ever)	radio<br>1 Yes<br>2 No<br>3 Truly unknown<br>4 Not asked / Not collected
	90	[stroke]	Stroke	radio<br>1 Yes<br>2 No<br>3 Truly unknown<br>4 Not asked / Not collected
	91	[stroke_ever]	Stroke (Ever)	radio<br>1 Yes<br>2 No<br>3 Truly unknown<br>4 Not asked / Not collected
	92	[priapism]	Priapism	radio<br>1 Yes<br>2 No<br>3 Truly unknown<br>4 Not asked / Not collected
	93	[priapism_ever]	Priapism (Ever)	radio<br>1 Yes<br>2 No<br>3 Truly unknown<br>4 Not asked / Not collected
	94	[renal_complications]	Renal Complications	radio<br>1 Yes<br>2 No<br>3 Truly unknown<br>4 Not asked / Not collected
	95	[renal_complications_ever]	Renal Complications (Ever)	radio<br>1 Yes<br>2 No<br>3 Truly unknown<br>4 Not asked / Not collected
	96	[jaundice]	Jaundice	radio<br>1 Yes<br>2 No<br>3 Truly unknown<br>4 Not asked / Not collected
	97	[jaundice_ever]	Jaundice (Ever)	radio<br>1 Yes<br>2 No<br>3 Truly unknown<br>4 Not asked / Not collected

	98	[abnorm_appetite]	Abnormal Appetite	radio<br>1 Yes<br>2 No<br>4 Not asked / Not collected
	99	[abnorm_stool_freq]	Abnormal Stool Frequency	radio<br>1 Yes<br>2 No<br>4 Not asked / Not collected
	100	[screening_complete]	Section Header: *Form Status*<br>Complete?	dropdown<br>0 Incomplete<br>1 Unverified<br>2 Complete
**Instrument: Demographics (demographics)**				
	101	[first_name1]	Section Header: *Patient Details*<br>First Name	text
	102	[medical_record_id]	Electronic Medical Record ID/ Patient Medical Record ID	text
	103	[data_source]	Where was the data collected ?	text
	104	[data_source_country]	Country where the data was collected ?	text<br>SELECT record | value FROM redcap_data WHERE project_id = 76 AND field_name = 'country' ORDER BY value
	105	[study_type]	Type of study the patient is enrolled in	dropdown<br>1 Meta-Analysis<br>2 Systematic Review<br>3 Randomized Controlled Trial<br>4 Cohort Study<br>5 Case-control Study<br>6 Cross-sectional study<br>7 Case Reports and Series<br>Ideas Editorials, Opinions<br>8 Animal Research Studies<br>9 Test-tube Lab Researc<br>10 Treatment Research<br>11 Prevention Research<br>12 Diagnostic Research<br>13 Screening Research<br>14 Quality of Life Research<br>15 Genetic studies<br>16 Epidemiological studies<br>17 Phase I Clinical trials<br>18 Phase II Clinical trials<br>19 Phase III Clinical trials<br>20 Phase IV Clinical trials
	106	[current_age_demo]	Current age in years<br>Current age from birth	calc<br>Calculation: datediff('today',[date_of_birth],"y","dmy")
	107	[registration_date]	Date on which the registration was done. (MM/DD/YYYY)	text (date_mdy)
	108	[interview_age]	Age in months at the time of the interview/test/sampling/imaging.	calc<br>Calculation: datediff([date_of_birth],[registration_date],"M","dmy")

	109	[current_martial_status_opposite_sex]	What is your current marital or cohabiting status?<br>*PX010902 | phenx_current_marital_status*	radio<br>1 Married<br>2 Not married but living together with a partner<br>3 Widowed<br>4 Divorced or annulled<br>5 Separated<br>6 Never been married
	110	[birthplace_location]	Where were you born?<br>*PX010201 | phenx_birthplace*	text
	111	[current_educational_attainment]	Section Header: *Socio-Demographics*<br>What is the highest grade or level of school you have completed or the highest degree you have received? [HAND CARD DMQ1. READ HAND CARD CATEGORIES IF NECESSARY. ENTER HIGHEST LEVEL OF SCHOOL.]<br>*PX011001 | phenx_current_educational_attainment*	dropdown<br>0 NEVER ATTENDED/KINDERGARTEN ONLY<br>1 1ST GRADE<br>2 2ND GRADE<br>3 3RD GRADE<br>4 4TH GRADE<br>5 5TH GRADE<br>6 6TH GRADE<br>7 7TH GRADE<br>8 8TH GRADE<br>9 9TH GRADE<br>10 10TH GRADE<br>11 11TH GRADE<br>12 12TH GRADE, NO DIPLOMA<br>13 HIGH SCHOOL GRADUATE<br>14 GED OR EQUIVALENT<br>15 SOME COLLEGE, NO DEGREE<br>16 ASSOCIATE DEGREE: OCCUPATIONAL, TECHNICAL, OR VOCATIONAL PROGRAM<br>17 ASSOCIATE DEGREE: ACADEMIC PROGRAM<br>18 BACHELOR'S DEGREE (EXAMPLE: BA, AB, BS, BBA)<br>19 MASTER'S DEGREE (EXAMPLE: MA, MS, MEng, MEd, MBA)<br>20 PROFESSIONAL SCHOOL DEGREE (EXAMPLE: MD, DDS, DVM, JD)<br>21 DOCTORAL DEGREE (EXAMPLE: PhD, EdD)<br>77 REFUSED<br>99 DON'T KNOW
	112	[income_currency]	Please select the income currency for your country.	dropdown<br>NGN Nigerian naira<br>USD US Dollar
	113	[an_fam_income_coded]	What was your best estimate of the total income of all family members from all sources, before taxes, in last year?<br>*PX011101 | phenx_annual_family_income*	text

	114	[current_employment_status]	We would like to know about what you do --are you working now, looking for work, retired, keeping house, a student, or what?<br>*PX011301 | phenx_current_employment_status*	dropdown<br>1 WORKING NOW<br>2 ONLY TEMPORARILY LAID OFF, SICK LEAVE OR MATERNITY LEAVE<br>3 LOOKING FOR WORK, UNEMPLOYED<br>4 RETIRED<br>5 DISABLED, PERMANENTLY OR TEMPORARILY<br>6 KEEPING HOUSE<br>7 STUDENT<br>8 OTHER (SPECIFY):
	115	[cur_employ_stat_specify]	current employment status, Other - specify<br>*PX011301 | phenx_current_employment_status*	text
	116	[description_female_210201]	This question is about the woman who functions as a mother in the respondent's household; she could be the biological mother, stepmother, foster mother, or adoptive mother or, perhaps, a grandmother or aunt. If there is no such woman, the question is skipped.<br>R=respondent<br>*PX0210201*	descriptive
	117	[answers_1_210201]	How far in school did she go?<br>*PX0210201*	radio<br>1 eighth grade or less<br>2 more than eighth grade, but did not graduate from high school<br>3 went to a business, trade, or vocational school instead of high school<br>4 high school graduate<br>5 completed a GED<br>6 went to a business, trade, or vocational school after high school<br>7 went to college, but did not graduate<br>8 graduated from a college or university<br>9 professional training beyond a four-year college or university<br>10 she never went to school<br>11 she went to school, but R doesn't know what level<br>12 R doesn't know if she went to school<br>13 refused<br>14 legitimate skip<br>15 don't know
	118	[description_male_210201]	This question is about the man who functions as a father in the respondent's household. If there is no such man, the question is skipped.<br>*PX0210201*	descriptive

	119	[answers_2_210201]	How far in school did he go?<br>*PX0210201*	radio<br>1 eighth grade or less<br>2 more than eighth grade, but did not graduate from high school<br>3 went to a business, trade, or vocational school instead of high school<br>4 high school graduate<br>5 completed a GED<br>6 went to a business, trade, or vocational school after high school<br>7 went to college, but did not graduate<br>8 graduated from a college or university<br>9 professional training beyond a four-year college or university<br>10 he never went to school<br>11 he went to school, but R doesn't know what level<br>12 R doesn't know if she went to school<br>13 refused<br>14 legitimate skip<br>15 don't know
	120	[health_insurance_coverage_type]<br>Show the field ONLY if:<br>[health_insurance_coverage_other] = '1'	What type of health insurance do you have?<br>*PX011502*	text
	121	[scd_martialstatus_primarycaregiver]	Marital status of primary caregiver<br>*PX0840101*	radio<br>UNDEFINED_CODE Married<br>UNDEFINED_CODE_1 Widowed<br>UNDEFINED_CODE_2 Separated<br>UNDEFINED_CODE_3 Divorced<br>UNDEFINED_CODE_4 Never married<br>UNDEFINED_CODE_5 Living with partner<br>UNDEFINED_CODE_6 Refused to answer<br>UNDEFINED_CODE_7 Unknown
	122	[scd_martialstatus_primarycaregiver_relationshipto_patient]	Relationship of primary caregiver to patient<br>*PX0840101*	radio<br>1 Mother<br>2 Father<br>3 Niece or nephew<br>4 Aunt or uncle<br>5 Cousin<br>6 Grandparent<br>7 Great grandparent<br>8 Foster parent<br>9 Other; explain below
	123	[scd_martialstatus_primarycaregiver_relationshipto_patientother]	Relationship of primary caregiver to patient<br>*PX0840101*	text<br>Other Other

	124	[demographics_complete]	Section Header: *Form Status*<br>Complete?	dropdown<br>0 Incomplete<br>1 Unverified<br>2 Complete
**Instrument: Anthropometrics (anthropometrics)**				
	125	[standing_ht_units]	Standing Height Units<br>*PX020703 | phenx_standing_height*	dropdown<br>ft Feet<br>in Inches<br>m Meters<br>Custom alignment: RH
	126	[standing_ht]	Standing Height<br>*PX020703 | phenx_standing_height*	text (number, Min: 0)
	127	[self_reported_ht_coded]	How tall {are you/is [participant]?} without shoes? ENTER HEIGHT IN FEET AND INCHES OR METERS AND CENTIMETERS<br>*PX020704 | phenx_selfreported_height*	radio<br>1 FEET AND INCHES<br>2 METERS AND CENTIMETERS<br>3 REFUSED<br>4 DON'T KNOW
	128	[self_reported_ht_proxy_name]	Proxy Name<br>*If applicable | | PX020704 | phenx_selfreported_height*	text
	129	[measured_wt_units]	Record Current Weight in Pounds or Kilograms.<br>*PX021501 | phenx_measured_weight*	dropdown<br>kg Kilograms<br>lbs Pounds<br>Custom alignment: RH
	130	[measured_wt]	Measured Weight<br>*PX021501 | phenx_measured_weight*	text (number, Min: 0)
	131	[self_reported_wt]	How much {do you/does the participant} weigh without clothes or shoes?<br>[If {you are/she is} currently pregnant, how much did {you/she} weigh before your pregnancy?]<br>*PX021502 | phenx_selfreported_weight*	text (number, Min: 0)
	132	[sp02]	Peripheral oxygen saturation (SpO2)	text
	133	[mid_upper_arm_circumferenc]	Mid-Upper Arm Circumference [MUAC] (Pediatrics)	text
	134	[anthropometrics_complete]	Section Header: *Form Status*<br>Complete?	dropdown<br>0 Incomplete<br>1 Unverified<br>2 Complete
**Instrument: Patient Follow Up (patient_follow_up)**				
	135	[visit_date]	Visit Date	text (date_dmy)
	136	[last_visit]	When was your last visit to the hospital or Sickle Cell Clinic	text (date_dmy)
	137	[how_many_crises_have_you_h]	How many crises have you had since your last visit?	text (number)
	138	[when_was_your_last_crisis]	When was your last crisis?	text (date_dmy)
	139	[weight]	Weight (kg)	text
	140	[height_length]	Height/Length (cm)	text
	141	[systol_blood_press]	Systolic blood pressure (mmHg)	text (integer)
	142	[diastol_blood_press]	Diastolic blood pressure (mmHg)	text (integer)
	143	[temp]	Temperature (Celcius)	text
	144	[spo2]	Peripheral oxygen saturation (SpO2)	text
	145	[muac]	Mid-Upper Arm Circumference [MUAC]	text

	146	[hb_quantitation]	Hb Quantitation (if available)	yesno<br>1 Yes<br>0 No
	147	[hbss]	HbSS %	text (number)
	148	[hbsc]	HbSC %	text (number)
	149	[hbf]	HbF %	text
	150	[hba2]	HbA2 %	text
	151	[hb_others]	Hb (others)	text
	152	[hb_g_dl]	Hb (g/dl)	text (number)
	153	[fbc_done]	FBC Done	yesno<br>1 Yes<br>0 No
	154	[date_of_fbc_result]	Date of FBC Result	text (date_dmy)
	155	[pcv]	PCV	text
	156	[wbc]	WBC	text
	157	[rbc]	RBC	text
	158	[mch]	MCH	text
	159	[reticulocyte_count]	Reticulocyte Count	text
	160	[mcv]	MCV	text
	161	[mchc]	MCHC	text
	162	[red_blood_cell_distribution_width]	Red Blood Cell Distribution Width	text
	163	[urea]	Urea	text
	164	[creatinine]	Creatinine	text
	165	[direct_bilirubin]	Direct Bilirubin	text
	166	[total_bilirubin]	Total Bilirubin	text
	167	[boold_transfusion]	Blood Transfusion	radio<br>1 Yes<br>2 No<br>4 Not asked / Not collected
	168	[units_transfused]<br>Show the field ONLY if:<br>[boold_transfusion] = '1'	Units of blood transfused	text
	169	[date_transfusion]<br>Show the field ONLY if:<br>[boold_transfusion] = '1'	Date of transfusion	text (date_ymd)
	170	[penicillin_prophylaxis]	Penicillin Prophylaxis	radio<br>1 Yes<br>2 No<br>4 Not asked / Not collected
	171	[folicacid_prophylaxis]	Folic Acid Prophylaxis	radio<br>1 Yes<br>2 No<br>4 Not asked / Not collected
	172	[antimal_prophylaxis]	Anti Malaria Prophylaxis	radio<br>1 Yes<br>2 No<br>4 Not asked / Not collected

	173	[hydroxyurea_use]	Using Hydroxyurea	radio<br>1 Yes<br>2 No<br>4 Not asked / Not collected
	174	[other_medications_taken]	Other Medications taken	text
	175	[pain_intensity_score]	Pain Intensity Score	dropdown<br>0 0<br>1 1<br>2 2<br>3 3<br>4 4<br>5 5<br>6 6<br>7 7<br>8 8<br>9 9<br>10 10
	176	[dactylitisfollowup]	Section Header: *Have you had any of the following after your last visit?*<br>Dactylitis	yesno<br>1 Yes<br>0 No
	177	[bone_pain_followup]	Bone Pain	yesno<br>1 Yes<br>0 No
	178	[hip_pain_followup]	Hip pain	yesno<br>1 Yes<br>0 No
	179	[chest_pain_followup]	Chest Pain	yesno<br>1 Yes<br>0 No
	180	[difficulty_in_walking_followup]	Difficulty in walking	yesno<br>1 Yes<br>0 No
	181	[difficulty_in_breathing_f]	Difficulty in Breathing	yesno<br>1 Yes<br>0 No
	182	[hospitalised_in_the_last_3]	Hospitalised in the last 30 days	yesno<br>1 Yes<br>0 No
	183	[malaria_followup]	Malaria	yesno<br>1 Yes<br>0 No
	184	[fever_followup]	Fever	yesno<br>1 Yes<br>0 No
	185	[anaemia_followup]	Anaemia	yesno<br>1 Yes<br>0 No

	186	[leg_ulcer_followup]	Leg ulcer	yesno<br>1 Yes<br>0 No
	187	[acute_chest_syndrome_f]	Acute chest syndrome	yesno<br>1 Yes<br>0 No
	188	[osteonecrosis_followup]	Osteonecrosis	yesno<br>1 Yes<br>0 No
	189	[stroke_followup]	Stroke	yesno<br>1 Yes<br>0 No
	190	[renal_complication_explain]	Renal complication explained by DR	yesno<br>1 Yes<br>0 No
	191	[protein_in_your_urine]	Protein in your urine	yesno<br>1 Yes<br>0 No
	192	[swollen_feet]	Swollen feet	yesno<br>1 Yes<br>0 No
	193	[morning_facial_swelling]	Morning facial swelling	yesno<br>1 Yes<br>0 No
	194	[dizziness_followup]	Dizziness	yesno<br>1 Yes<br>0 No
	195	[headache_followup]	Headache	yesno<br>1 Yes<br>0 No
	196	[swelling_followup]	Swelling	yesno<br>1 Yes<br>0 No
	197	[where]	Where?	text
	198	[priapism_followup]	Priapism	yesno<br>1 Yes<br>0 No
	199	[jaundice_followup]	Jaundice	yesno<br>1 Yes<br>0 No
	200	[exertional_dyspnea_exertio]	Exertional dyspnea Exertional dyspnea	yesno<br>1 Yes<br>0 No
	201	[abnormal_gait_followup]	Abnormal GAIT	yesno<br>1 Yes<br>0 No
	202	[other_pain_site_followup]	Other Pain Site	text

	203	[abnormal_appetite_followup]	Abnormal Appetite	text
	204	[stool_frequency_per_day]	Stool Frequency per day?	dropdown<br>1 | 1<br>2 | 2<br>3 | 3<br>4 | 4<br>5 | 5<br>6 | 6
	205	[using_an_android_phone]	Are you using an Android Phone?	yesno<br>1 | Yes<br>0 | No
	206	[are_you_on_social_media]	Are you on Social Media?	yesno<br>1 | Yes<br>0 | No
	207	[which_social_media]	Which Social Media	checkbox<br>1 | which_social_media___1 | Facebook<br>2 | which_social_media___2 | Twitter<br>3 | which_social_media___3 | Instagram<br>4 | which_social_media___4 | WhatsApp<br>5 | which_social_media___5 | Snapchat<br>6 | which_social_media___6 | Youtube<br>7 | which_social_media___7 | TikTok<br>8 | which_social_media___8 | Telegram
	208	[follow_up_date]	Follow Up Date	text (date_dmy)
	209	[relocated_to_a_new_site]	Patient Relocated to a New Site	yesno<br>1 | Yes<br>0 | No
	210	[please_specify_site_na]	Please specify the site name	text
	211	[lost_to_follow_up]	Lost to Follow-Up	yesno<br>1 | Yes<br>0 | No
	212	[date_of_last_to_follow_up]	Date of Last to Follow-Up	text (date_dmy)
	213	[patient_is_late]	Patient is Late	yesno<br>1 | Yes<br>0 | No
	214	[date_of_demise]	Date of Demise	text (date_dmy)
	215	[patient_follow_up_complete]	Section Header: *Form Status*<br>Complete?	dropdown<br>0 | Incomplete<br>1 | Unverified<br>2 | Complete
